# Improving implementation of Enhanced Recovery After Surgery (ERAS) to increase timeliness of recovery after cardiac surgery: a quality improvement project

**DOI:** 10.1136/bmjoq-2025-003612

**Published:** 2026-02-02

**Authors:** Adarsh Arun Menon, Rahul Mudannayake, Jonathan Bland, Caroline Gerrard, Matthew Petty, Nicola Jones

**Affiliations:** 1 University of Cambridge Department of Medicine, Cambridge, UK; 2 Department of Anaesthesia, Princess Alexandra Hospital, Woolloongabba, Queensland, Australia; 3 Royal Papworth Hospital NHS Foundation Trust, Cambridge, UK; 4 University of Cambridge School of Clinical Medicine, Cambridge, UK

**Keywords:** Critical care, PDSA, Performance measures, Quality improvement

## Abstract

**Background:**

The COVID-19 pandemic has exacerbated the backlog of elective surgeries across the National Health Service (NHS). This is particularly critical for patients awaiting cardiac surgery, where even short delays can lead to disease progression and increased risk of complications. Enhanced Recovery After Surgery (ERAS) programmes aim to optimise recovery and reduce length of stay, yet their implementation in cardiac surgery remains inconsistent. This quality improvement project sought to improve the implementation of postoperative ERAS principles to increase the timeliness of recovery and enhance intensive care unit (ICU) capacity.

**Methods:**

Time-directed ERAS goals were developed, and a phased educational intervention was implemented through four Plan–Do–Study–Act cycles: (1) introductory teaching and baseline data collection, (2) development of a tool within the electronic patient record to promote real-time implementation of ERAS goals and enable continuous performance monitoring, (3) introduction of an e-learning module and (4) targeted educational interventions. Outcome measures included time to achieve ERAS goals and the proportion of postoperative patients clinically ready for discharge to the ward within 24 and 48 hours. Balancing measures included reintubation and ICU readmission rates.

**Results:**

Implementation of the phased educational intervention led to a sustained reduction in the time required to achieve ERAS goals. The proportion of patients clinically ready for discharge to the ward within 24 and 48 hours increased by 15.6% and 18.0%, respectively, exceeding the project’s 5% target. No increase in reintubation or ICU readmission rates was observed, indicating that improvements were achieved safely.

**Conclusions:**

Implementing time-directed ERAS goals through a phased educational intervention increased the timeliness of post-operative recovery after cardiac surgery. This approach has the potential to improve patient flow, enhance ICU capacity and support wider efforts to address elective cardiac surgery backlogs across the NHS.

**WHAT IS ALREADY KNOWN ON THIS TOPIC**

- Enhanced Recovery After Surgery (ERAS) programmes are known to improve postoperative recovery and decrease length of stay; however, their implementation in cardiac surgery is variable, contributing to delays in achieving recovery milestones and limiting intensive care unit (ICU) capacity.

**WHAT THIS STUDY ADDS**

- This project shows that operationalising ERAS principles into time-directed key performance indicators can reduce variation in practice and produce sustained improvements in recovery timeliness, thereby addressing a recognised gap between ERAS guidelines and routine postoperative care. This is greatly facilitated by a digital mechanism for data collection integrated seamlessly with existing workflows, permitting close and continuous monitoring of targeted interventions in a high-acuity cardiac ICU setting.

**HOW THIS STUDY MIGHT AFFECT RESEARCH, PRACTICE OR POLICY**

- This work offers a practical, scalable model for embedding ERAS principles into postoperative cardiac surgical care to improve patient flow and support system-wide efforts to reduce backlogs for elective surgery.

## Introduction

### Problem

The combination of a growing and ageing population, coupled with the significant disruption to the National Health Service (NHS) caused by the COVID-19 pandemic, has resulted in a significant backlog of elective surgeries. This has led to record-high waiting lists and prolonged waiting times for patients.^1^ Consequently, restoring elective surgeries and reducing backlogs are top priorities for the NHS.^2^

These disruptions are particularly critical for those awaiting cardiac surgery, as even short delays can lead to the progression of heart conditions, increasing the risk of complications and life-threatening events.^3 4^ The British Heart Foundation has emphasised that cardiac care in the UK has reached a tipping point, with treatment delays jeopardising outcomes and contributing to increased morbidity and mortality among individuals with serious heart conditions.^5^

Timely intervention is therefore essential to avoid preventable deterioration, making the recovery of elective cardiac surgeries an urgent priority within the broader NHS recovery agenda.^5^

### Available knowledge

A potential approach to addressing this backlog and restoring elective surgeries is to improve patient flow through the implementation of Enhanced Recovery After Surgery (ERAS) programmes. Developed in the late 1990s by Dr Henrik Kehlet in the context of colorectal surgery, they aim to reduce the physiological and psychological stress of surgery via structured, patient-centred, multidisciplinary approaches spanning the preoperative, intraoperative and postoperative phases.^6^

The preoperative phase focuses on optimising patients’ readiness for surgery through education, managing underlying health conditions and improving nutrition and physical activity. During the intraoperative phase, physiological stress is minimised through refined surgical techniques, tailored anaesthesia and precise fluid management. The postoperative phase then promotes a swift return to normal function by emphasising early mobilisation, effective pain control and the resumption of normal diet.

ERAS programmes have been shown to enhance recovery and accelerate the return to normal functioning, reducing the length of stay (LoS) without increasing complications.^6^ This improvement in patient flow may increase capacity for elective procedures, suggesting ERAS as a potential approach to addressing surgical backlogs and supporting the restoration of elective services within the NHS.

### Rationale

Although established for over two decades in other surgical disciplines, the adoption of ERAS programmes in cardiac surgery is relatively recent, with guidelines for perioperative management in elective cardiac procedures first published in 2019 (and updated in 2024).^7 8^ However, implementation remains variable, as standardising protocols poses challenges due to the complexity of cardiac surgery. Nonetheless, evidence suggests ERAS in cardiac surgery enhances recovery, shortens LoS and reduces postoperative complications, as in other specialties.^9^,^12^

These outcomes support the potential of ERAS to help address surgical backlogs and restore elective services in the cardiac setting. A key constraint on the ability to proceed with elective cardiac surgery is the availability of a specialised postoperative recovery bed. Recognising this, we sought to evaluate whether implementing the postoperative phase of ERAS in a structured, standardised manner could enhance recovery, reduce LoS and increase capacity to support further elective cardiac surgeries.

### Specific aims

We undertook this quality improvement project (QIP) to improve implementation of postoperative ERAS principles and to evaluate whether this could enhance the timeliness of recovery after cardiac surgery.

We aimed to achieve a 5% increase in the proportion of postoperative cardiac surgery patients medically fit for transfer from the cardiothoracic intensive care unit (ICU) to the ward within 24 and 48 hours of surgery over a 12-month period. We considered this target achievable, realistic and meaningful—even a modest improvement in timely discharge readiness would increase patient flow through the ICU—potentially expanding capacity for 50–100 additional elective cases annually within a caseload of approximately 1000–2000 patients.

## Methods

### Context

As a leading tertiary referral centre for heart and lung disease, our institution is among the highest-volume adult cardiac surgery centres in the UK.^13^ During the COVID-19 pandemic, it was among the few centres providing extracorporeal membrane oxygenation for severe respiratory failure, which caused substantial disruption to elective services, underscoring the need to improve patient flow and restore elective surgical activity.

Postoperative care follows guidelines that incorporate ERAS principles and are intended to apply to all surgical patients, including those who are elderly, have significant comorbidities or undergo complex procedures. However, variability in their application during the immediate postoperative period was recognised by senior clinicians, highlighting opportunities to improve practice. Given post-operative cardiac surgery patients account for ~50%–60% of our ICU admissions, there was a clear opportunity to improve practice and increase effective ICU capacity through more consistent implementation of ERAS principles.

### Interventions

#### Development of time-directed ERAS key performance indicators

The QIP was undertaken by a multidisciplinary core team comprising senior anaesthetists/intensivists, the ICU lead nurse, electronic patient record (EPR) developers, and a medical student. In consultation with the wider multidisciplinary team (MDT), the QIP team identified uncertainty in applying ERAS principles within routine postoperative critical care as a barrier to consistent practice.

Therefore, the QIP team worked with the senior clinicians to operationalise existing ERAS guidelines into a set of specific, time-directed key performance indicators (KPIs) to support timely recovery after cardiac surgery. These were informed by evidence review, local practice insights and clinical experience. The agreed KPIs included: stopping sedation within 1 hour, achieving spontaneous breathing within 2 hours, extubation by 4 hours, initiating oral intake within 6 hours, mobilisation within 12 hours, chest drain removal by 24 hours, and clinically ready for discharge to the ward to the ward within 24–48 hours.^14^ Clinical readiness for ward discharge was selected rather than actual discharge, as it is more sensitive to the impact of ERAS implementation and timeliness of recovery, and less affected by organisational factors such as ward bed availability and discharge coordination.

#### Implementation of time-directed ERAS KPIs

To embed these KPIs into everyday clinical practice, the QIP team designed and implemented a phased educational intervention delivered across four iterative phases, refined through multidisciplinary feedback and serial data analysis. Each phase was designed to progressively strengthen MDT understanding and implementation of ERAS principles and time-directed goals in the ICU.

- **Phase 1:** An introductory 1-hour teaching session was delivered to the MDT, introducing ERAS principles and the new time-directed KPIs. ICU nurses also completed a paper-based form for each postoperative cardiac surgery patient, recording the time taken to achieve each KPI and reasons for any delays, to establish baseline performance and identify barriers to timely recovery.
- **Phase 2:** Findings from phase 1 informed the development of a structured tool within the EPR to prompt real-time implementation of the time-directed KPIs. The tool enabled nurses to record time taken to achieve KPIs and document reasons for any delays, using predefined categories or free-text entries, supporting consistent documentation and continuous monitoring of performance.
- **Phase 3:** An e-learning module was co-created by the QIP team and MDT to provide accessible, ongoing training on ERAS and the KPIs. It reinforced key elements of post-operative recovery and was supported by facilitated teaching sessions to consolidate understanding, address residual uncertainties and maintain engagement across staff groups.
- **Phase 4:** Targeted educational interventions were introduced to address ERAS goals that remained inconsistently achieved. They were determined post hoc, guided by analysis of EPR data and MDT feedback. These interventions were iteratively refined through review and collaborative discussion to sustain improvement and optimise implementation. They involved bitesize learning delivered during daily safety briefings and weekly MDT teaching, supported by relevant educational resources in staff areas.

A multimodal engagement strategy was used to ensure MDT awareness, participation and continuity throughout the project: information about the QIP was shared through posters in clinical areas, email updates and integrated into daily handovers and weekly meetings. Regular updates on progress and performance data were provided, with feedback actively sought to inform ongoing refinement. This collaborative, transparent approach aimed to improve implementation of the time-directed KPIs and embed them into consistent clinical practice.

### Study of the interventions

To assess the impact of the phased educational intervention, we applied the Institute for Healthcare Improvement’s Model for Improvement, using a series of four Plan–Do–Study–Act (PDSA) cycles,^15^ corresponding to the aforementioned intervention phases.

Achievement of KPIs and reasons for delays were continuously monitored to assess trends in relation to each phase of the educational intervention. Temporal changes in the percentage of postoperative cardiac surgery patients medically fit for step-down to the ward within 24 and 48 hours of surgery were compared between the start and the end of the project.

This approach aligned directly with the project’s specific aim and overall intention to improve the implementation of ERAS principles and thereby increase the timeliness of recovery after cardiac surgery through phased educational interventions.

### Measures

#### Outcome measures

Two primary outcome indicators were assessed:

1. time taken to achieve each KPI and the clinical/organisational reasons for any delays.
2. percentage of postoperative cardiac surgery patients medically fit for transfer from the ICU to the ward within 24 and 48 hours of surgery. These measures assessed how consistently ERAS goals were achieved within expected time frames, why delays occurred, and whether improved implementation was associated with increased timeliness of postoperative recovery.

#### Process measures

Engagement with the intervention was assessed by monitoring completion of the EPR tool. For each KPI, the number of completed entries was expressed as a percentage of all eligible cases, providing an indicator of staff participation and consistency in applying ERAS principles.

#### Balancing measures

To monitor for unintended consequences of accelerating postoperative care, we tracked reintubation and ICU readmission rates as balancing measures. These metrics ensured efforts to improve discharge readiness and KPI achievement did not lead to patient complications or compromise overall care.

### Analysis

Data for analysis were collected from two sources aligned with the intervention phases: paper-based forms completed by ICU nurses during phase 1 and data extracted from the EPR tool during phases 2–4. When forms were incomplete, we conducted a hand search of the EPR to determine the time to KPI achievement and the reasons for any delays.

Quantitative data on the time taken to achieve each KPI were plotted on run charts to analyse temporal variation. Run charts were selected as the project was originally expected to run for 12 months and therefore not anticipated to yield enough data points for more sophisticated statistical process control methods. They also provided a clear, theory-informed method to detect non-random variation and identify changes temporally associated with interventions across PDSA cycles. The first 3 months of data were used as a baseline to establish the median line, providing a reference point to identify special cause variation (SCV). Run charts were interpreted according to the four established rules for identifying SCV: (1) a shift (6+ consecutive points on one side of the median), (2) a trend (5+ consecutive points all increasing or decreasing), (3) too many/few runs (outside the expected range) and (4) an astronomical data point.^16^

Qualitative data on reasons for delays were grouped into predefined categories and summarised using Pareto charts to identify the most frequent and influential factors. This approach highlighted the key factors to inform iterative refinement of the phased educational intervention.

Changes in the percentage of patients medically fit for step-down to the ward within 24 and 48 hours were compared between the start and end of the project. Descriptive statistics and two-tailed t-tests were used to determine whether observed differences were statistically significant.

Completion rates of the EPR tool and trends in reintubation and ICU readmission were analysed descriptively to assess engagement, data completeness and patient safety.

All analyses were conducted using R (V.4.4.0).^17^

Patient-identified priorities shaped this QIP, particularly the expectation for timely surgery, rapid recovery and an uncomplicated postoperative course. These perspectives informed the selection of outcomes/KPIs relevant to patients. Active dissemination of results via internal networks and social media will support public engagement and awareness of care pathway improvements.^18^

## Results

The project was initially designed as a 12-month initiative but was extended owing to real-world delays in the development, implementation and refinement of the EPR tool. It ran from June 2022 to April 2024, progressing through four iterative phases aligned with PDSA cycles: PDSA-1 (June 2022), PDSA-2 (December 2022–February 2023), PDSA-3 (April–August 2023) and PDSA-4 (September 2023–April 2024). Results are presented according to these four cycles, with outcome, process and balancing measures analysed to assess change over time.

### Outcome measures

**Figure 1:**
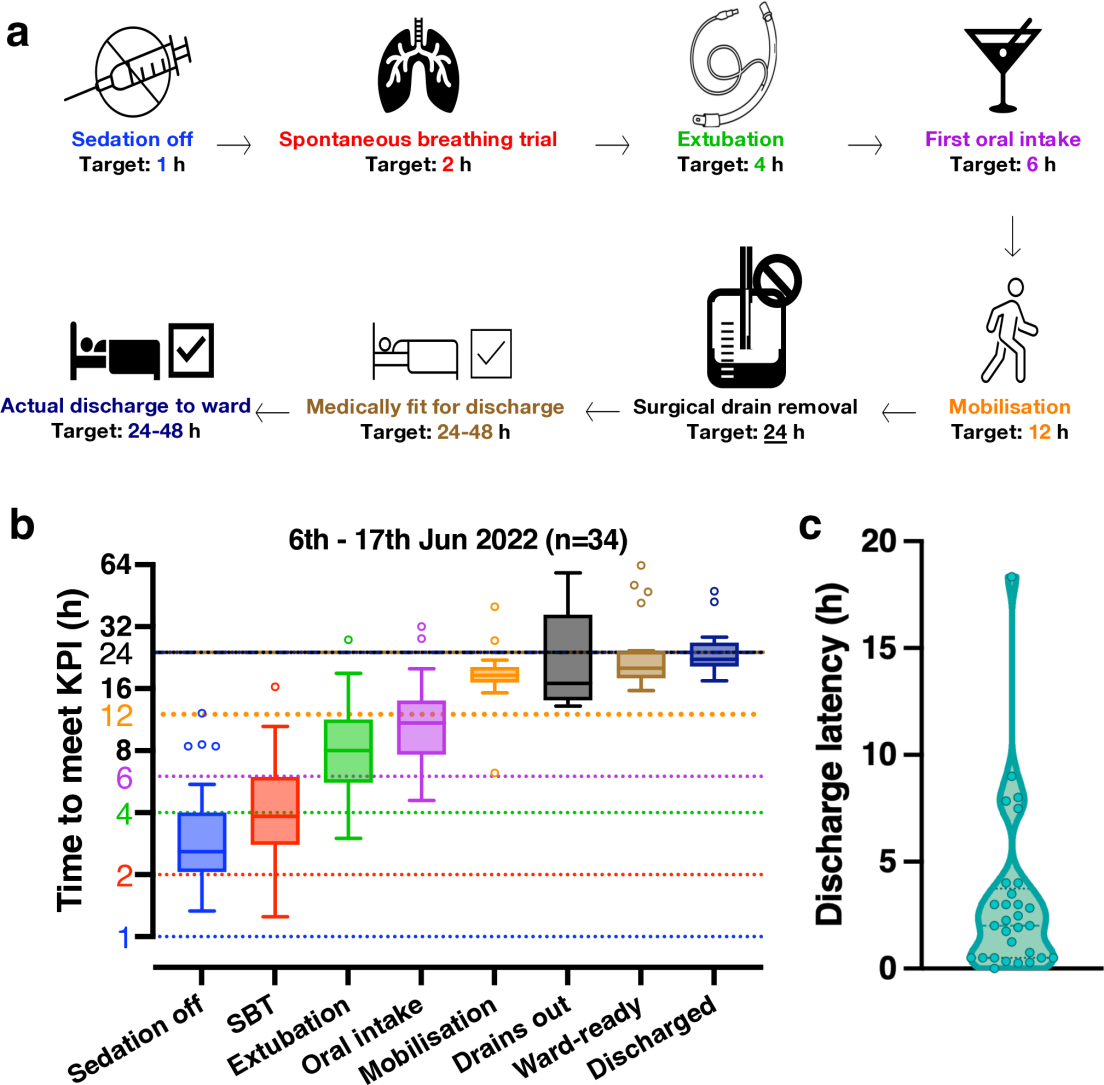
**Defining the time-directed key performance indicator (KPI)-based protocol.** (a) Flow chart illustrating the aspirational elective cardiac surgical patient journey within our institution’s intensive care unit (ICU), after arrival from the operating theatre through to ICU discharge. (b) Boxplots for each of the specified KPIs during the baseline data collection period. Dotted lines indicate local targets for meeting each performance indicator and are colour-coded to match the boxplot of their respective performance indicator. Note the logarithmic scale for the y-axis. (c) Violin plot of discharge latency, that is, the time lag between a patient being deemed ready for discharge to the ward and actually being discharged from ICU. Dotted lines represent quartiles and median. KPI, key performance indicator; SBT, spontaneous breathing trial***.***

Between 6 June 2022 and 17 June 2022 (PDSA-1), ICU nurses completed 34 paper-based forms for postoperative cardiac surgery patients, documenting the time taken to achieve each KPI and reasons for any delays (figure 1). Review of these forms by the QIP team provided quantitative and qualitative insights into current practice.

Analysis demonstrated the time taken to achieve all KPIs exceeded the agreed time-directed targets, except for drain removal and clinical readiness for ICU discharge. Median times were 2.6 hours for stopping sedation (target 1 hour), 3.8 hours for achieving spontaneous breathing (target 2 hours), 8.0 hours for extubation (target 4 hours), 10.9 hours for initiating oral intake (target 6 hours), 18.6 hours for mobilisation (target 12 hours), 17 hours for drain removal (target 24 hours) and 20.1 hours for clinical readiness for ICU discharge (target 24–48 hours).

Qualitative analysis identified clinical and organisational factors contributing to these delays, including excessive bleeding, cardiovascular instability, patient drowsiness, limited staffing and ward-bed availability (online supplemental figure 1). Comparison of nurse-reported and objectively reviewed data showed that delays were often under-recognised at the bedside, particularly for sedation off, extubation, oral intake and mobilisation (online supplemental table 1).

These findings indicated scope for improvement and informed the design of the EPR tool introduced in PDSA-2 (online supplemental figure 2), which served as both a prompt to reinforce ERAS goals and a mechanism for monitoring performance.

Pareto chart analysis of data extracted from the EPR tool between December 2022 and February 2023 (PDSA-2) (online supplemental figure 3) identified that a small number of factors accounted for most delays in achieving KPIs. Clinical factors predominated, with patient drowsiness, poor respiratory drive and cardiovascular instability representing the most frequent contributors to delays in sedation cessation, extubation and initiation of oral intake. For later-phase goals such as mobilisation and drain removal, pain, nausea/vomiting and surgeon preference for drain retention were the main causes.

Many of these clinical causes were potentially modifiable through the application of ERAS principles—specifically optimising sedation management, analgesia, antiemetic use and early drain removal. These insights informed the design of the e-learning module implemented in PDSA-3 and the targeted educational interventions delivered in PDSA-4, which reinforced these principles to reduce clinically driven delays.

**Figure 2:**
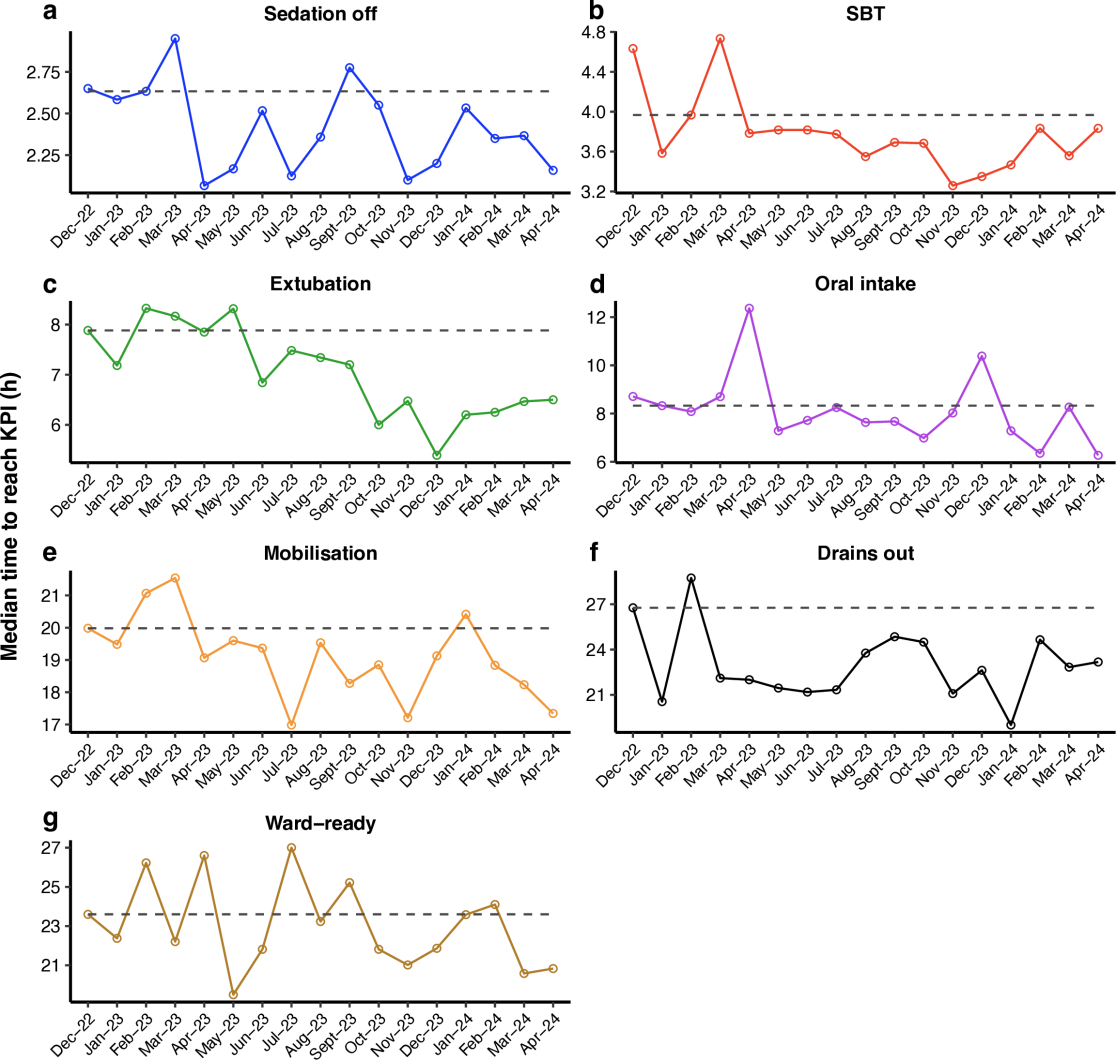
**Run-chart KPI data since the implementation of the EPR-based form. **Panels illustrate median time, measured from the point of admission to ICU, to (a) sedation off, (**b**) spontaneous breathing trial, (**c**) extubation, (**d**) oral intake, (**e**) mobilisation, (**f**) removal of surgical drains, (**g**) medically fit for discharge to ward. In each panel, the grey dashed line represents the median line, calculated from the first 3 months of data. Evidence of special cause variation was observed in a–f. EPR, electronic patient record; ICU, intensive care unit; KPI, key performance indicator; SBT, spontaneous breathing trial*.*

Quantitative analysis of data extracted from the EPR tool between December 2022 and April 2024 showed progressive reductions in the average time to achieve each ERAS goal across PDSA cycles 2–4. Run chart analysis (figure 2) demonstrated sustained downward shifts in time to completion across all goals except clinical readiness for ward discharge. Evidence of SCV was identified in six of the seven measures. Sedation off, spontaneous breathing trial, extubation, oral intake, mobilisation and drain removal each displayed 6+consecutive data points below the baseline median and/or too few runs, fulfilling established run chart rules for non-random variation.

Shifts observed from April 2023 corresponded to the introduction of the e-learning module (PDSA-3), which was associated with initial improvements followed, in some measures, by temporary deterioration and prolongation in times to goal achievement. This informed the development of targeted educational interventions (PDSA-4). Subsequent improvements were again noted from September 2023 for sedation cessation, from December 2023 for oral intake, and from January 2024 for mobilisation, consistent with the effects of the intensified educational focus.

By the end of the project, all KPI times were shorter than at baseline, except for clinical readiness for ICU discharge. The shortest median times achieved were 2.1 hours for stopping sedation, 3.3 hours for spontaneous breathing, 5.4 hours for extubation, 6.3 hours for initiating oral intake, 17.0 hours for mobilisation and 19.0 hours for chest drain removal.

**Figure 3:**
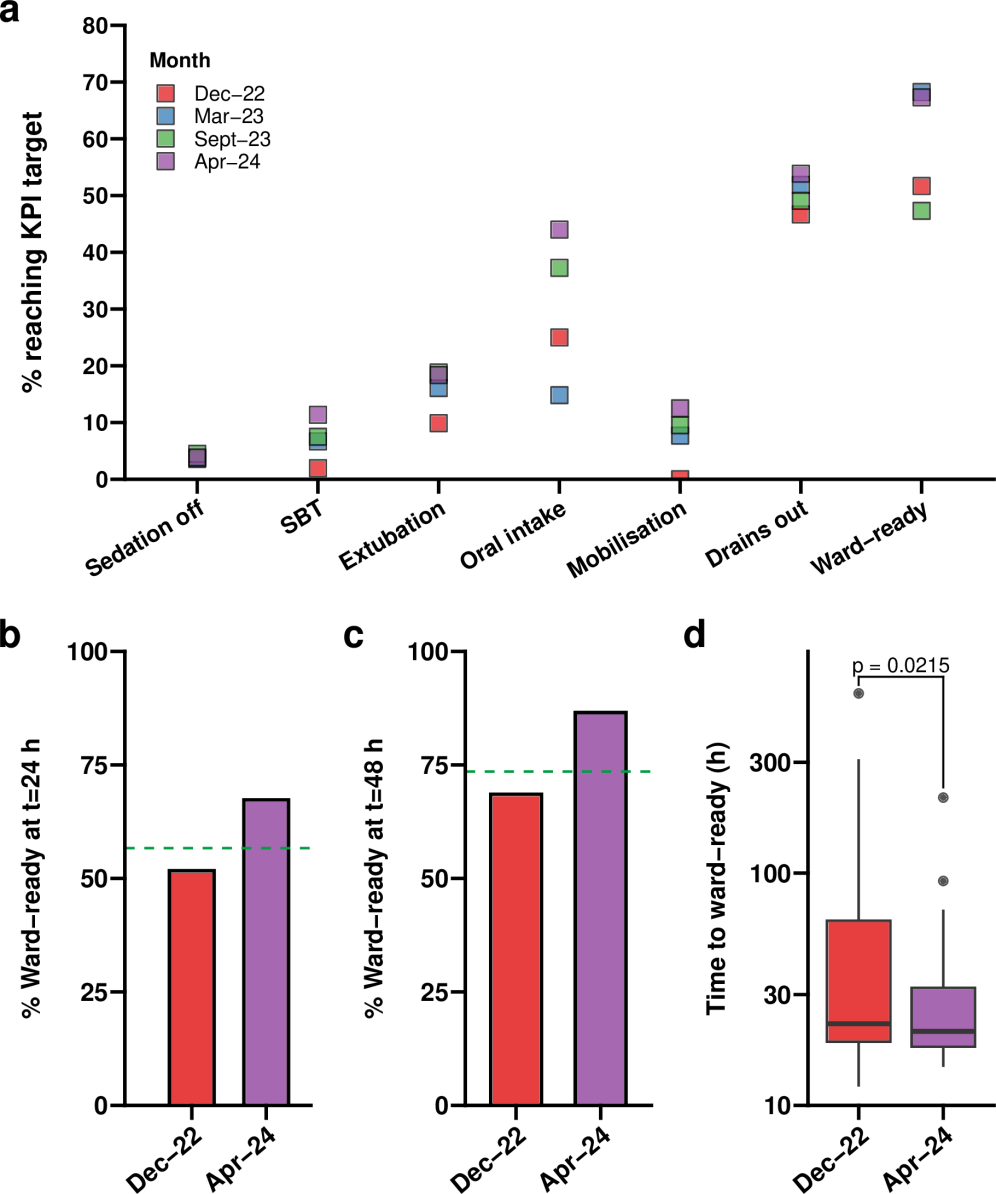
**Quantifying rates of key performance indicator (KPI) targets being met following implementation of QI interventions.** (a) Percentage of patients meeting each KPI target at baseline (December 2022), once the EPR form had been well-established into routine practice (March 2023), after implementing the educational interventions (September 2023) and at the end of the project (April 2024). (b–c) Comparison of the percentage of patients deemed medically fit for discharge to the ward (‘ward-ready’) at baseline and at the end of the project within 24 hours (b) and within 48 hours (c). Dashed green lines represent the threshold required to meet the target of a 5% increase in the proportion of post-operative cardiac surgery patients medically fit for transfer from the cardiothoracic intensive care unit (ICU) to the ward set out at the start of the project. (d) Boxplot of the time taken to be deemed ward ready at baseline and at the end of the project. p=0.0215 by two-tailed Student’s t-test. EPR, electronic patient record; SBT, spontaneous breathing trial.

In contrast, median times for clinical readiness for ICU discharge remained stable over the project, with no SCV observed. However, the proportion of patients meeting this criterion within 24 and 48 hours increased by 15.6% and 18.0%, respectively (figure 3).

No changes in patient casemix, surgical or anaesthetic practice, or other contextual factors were identified during this period, supporting the inference that improvements in timeliness and discharge readiness were attributable to the phased educational interventions.

### Process measures

Completion rates for the EPR tool broadly waned as the QIP progressed, ranging from 97% (sedation off/extubation, July 2023) to 10% (mobilisation, February 2024). Observational feedback suggested that ERAS-related actions were often performed but not consistently recorded.

### Balancing measures

Reintubation and ICU readmission rates remained stable throughout the QIP, showing no increase compared with the preintervention period, indicating the observed improvements in timeliness and discharge readiness were achieved without compromising patient safety or clinical outcomes.

## Discussion

We undertook this QIP to strengthen the implementation of ERAS principles and increase the timeliness of recovery after cardiac surgery. It operationalised ERAS principles into specific, time-directed goals and was delivered through an iterative, phased educational approach comprising four PDSA cycles.

The intervention was associated with progressive and sustained reductions in the time taken to achieve predefined KPIs, alongside a marked increase in the proportion of patients achieving clinical readiness for ICU discharge within 24 and 48 hours, exceeding the predefined ‘SMART’ target of a 5% improvement. All gains were sustained without any increase in reintubation or ICU readmission rates, indicating that improvements were achieved safely.

This QIP demonstrates a structured and reproducible approach to improving ERAS implementation and enhancing the timeliness of postoperative recovery. By increasing clinical readiness for ward discharge, the intervention has the potential to release ICU capacity to support elective cardiac surgery and address service backlogs. Based on the observed 15% increase in readiness within 24 hours in a caseload of approximately 1000 procedures per year, this improvement could translate to capacity for up to 150 additional elective operations annually.

Key strengths included the iterative refinement of educational interventions, guided by multidisciplinary feedback and data-driven analysis, to optimise implementation of ERAS goals. Its application across all postcardiac surgery patients—including those of advanced age, with multiple comorbidities, or undergoing complex procedures—supports the scalability and transferability of this approach across diverse cardiac surgical settings.

### Interpretation

These findings are consistent with published evidence indicating that implementing ERAS principles into postoperative cardiac surgery protocols can effectively reduce ICU LoS. Earlier cessation of sedation is likely to facilitate the timely achievement of key recovery milestones including spontaneous breathing, extubation, resumption of oral intake and mobilisation. Supporting this, qualitative data from our QIP identified patient drowsiness as the most frequent factor contributing to delays in achieving time-directed ERAS goals.

Existing literature also highlights that educational interventions which strengthen staff understanding and ownership of ERAS principles enhance their integration into clinical practice.^19^ Furthermore, evidence suggests that setting realistic, locally relevant, time-directed targets can help standardise practice and reduce variation.^20^ Our approach to operationalising ERAS guidelines into specific, measurable KPIs provided a clear framework to improve implementation and increase the timeliness of recovery.

In addition to improving the implementation of ERAS principles, the EPR tool developed during this project provided a mechanism for capturing data relevant to nationally reported quality indicators. Specifically, it supported reporting against the NHS Commissioning for Quality and Innovation target introduced in 2023, which requires ≥80% of surgical inpatients to be DRinking, Eating and Mobilising (DREaMing) within 24 hours of surgery.^21^ This alignment with national quality priorities further reinforces the broader relevance and scalability of the intervention.

Although the time taken to achieve most ERAS goals shortened across the project, the targeted time frames were not fully met, suggesting that educational interventions alone may be insufficient. Some delays, such as those related to bleeding or haemodynamic instability, are not readily influenced by education and may require broader changes in clinical practice.

The one factor that did not change was time to clinical readiness for ICU discharge. This likely reflects how readiness was assessed—not solely on clinical criteria but also on the appropriateness of transfer, which was typically reviewed only during daytime hours. Ultimately, however, the realisation of ICU capacity depends not only on timelier recovery but also on actual ward transfer, which is constrained by organisational factors such as bed availability, staffing levels and discharge coordination.

Further improvements may be achieved by extending ERAS implementation beyond the immediate postoperative period to encompass the entire surgical pathway. Earlier optimisation and reduced intraoperative stress could enhance recovery, while improved ward flow and discharge processes would help translate these patient-level gains into system-level benefits—supporting timelier recovery, optimising the use of ICU capacity and enabling increased elective surgical throughput to help address the backlog.

The project required minimal financial investment, relying primarily on existing staff and resources. For long-term sustainability, ongoing investment in dedicated educational and digital support may be warranted, offset by potential gains from reduced ICU LoS, improved patient flow and expanded elective surgical capacity.

### Limitations

This QIP was conducted in a single specialised tertiary referral centre, which may limit its generalisability to other settings. However, as our institution follows widely adopted approaches and most of our caseload involved common cardiac surgeries, the findings are likely applicable to other cardiac surgical contexts.

A further limitation of this QIP was its reliance on an EPR-based tool, which served both as a prompt for ICU nurses to implement ERAS principles and as a data collection tool to monitor outcomes and guide subsequent QIP phases. In settings without similar EPR capabilities or those reliant on paper-based systems, alternative methods may need to be developed to achieve similar functionality.

Variable completion rates of the EPR-based tool represented another limitation, resulting in incomplete process measures. Despite this, observable changes in practice—such as earlier cessation of sedation—occurred in temporal association with the phased educational intervention and in the absence of other identifiable contextual factors, suggesting improvements were attributable to the intervention itself. Incomplete documentation and variability in process measures are well-recognised challenges in QIPs.^22^ The recent establishment of a dedicated post-cardiac surgery ICU team provides an opportunity for further targeted educational interventions to strengthen compliance and data completeness.

### Conclusions

This QIP demonstrates the utility of operationalising ERAS principles into specific, time-directed goals and implementing them through a phased educational intervention. The project achieved reductions in the time required to meet KPIs and significantly increased the proportion of postoperative patients clinically ready for discharge to the ward within both 24 and 48 hours, without any increase in complications. Sustainability was facilitated by embedding an electronic tool within the EPR, serving as both an educational prompt for ICU nurses and a data collection tool, enabling continuous monitoring and iterative refinement.

The intervention was applied across a broad caseload of cardiac surgical procedures, suggesting its potential generalisability to other cardiac surgical settings. However, reliance on an electronic tool highlights the need for alternative approaches in environments using paper-based systems or different EPR platforms.

Future work should explore whether these improvements translate into greater patient flow, increased ICU capacity and reduced elective surgery backlogs. Greater impact may be achieved by embedding ERAS principles across the entire surgical pathway—including preoperative and intraoperative phases—as part of an overarching quality improvement strategy. National audits of cardiac surgery outcomes could stratify by ERAS implementation to evaluate system-wide benefits and support the integration of these principles into routine practice.

## Supplementary material

10.1136/bmjoq-2025-003612 online supplemental figure 1

10.1136/bmjoq-2025-003612 online supplemental figure 2

10.1136/bmjoq-2025-003612 online supplemental figure 3

10.1136/bmjoq-2025-003612 online supplemental table 1

## Data Availability

Data are available on reasonable request.
