# Supplementary material for: Improving implementation of Enhanced Recovery After Surgery (ERAS) to increase timeliness of recovery after cardiac surgery: a quality improvement project
Source: BMJ Open Qual. 2026 Feb 2;15(1):e003612. doi: 10.1136/bmjoq-2025-003612 (PMC12878191; doi:10.1136/bmjoq-2025-003612)
Supplement: online supplemental figure 1 [file bmjoq-15-1-s001.pdf]

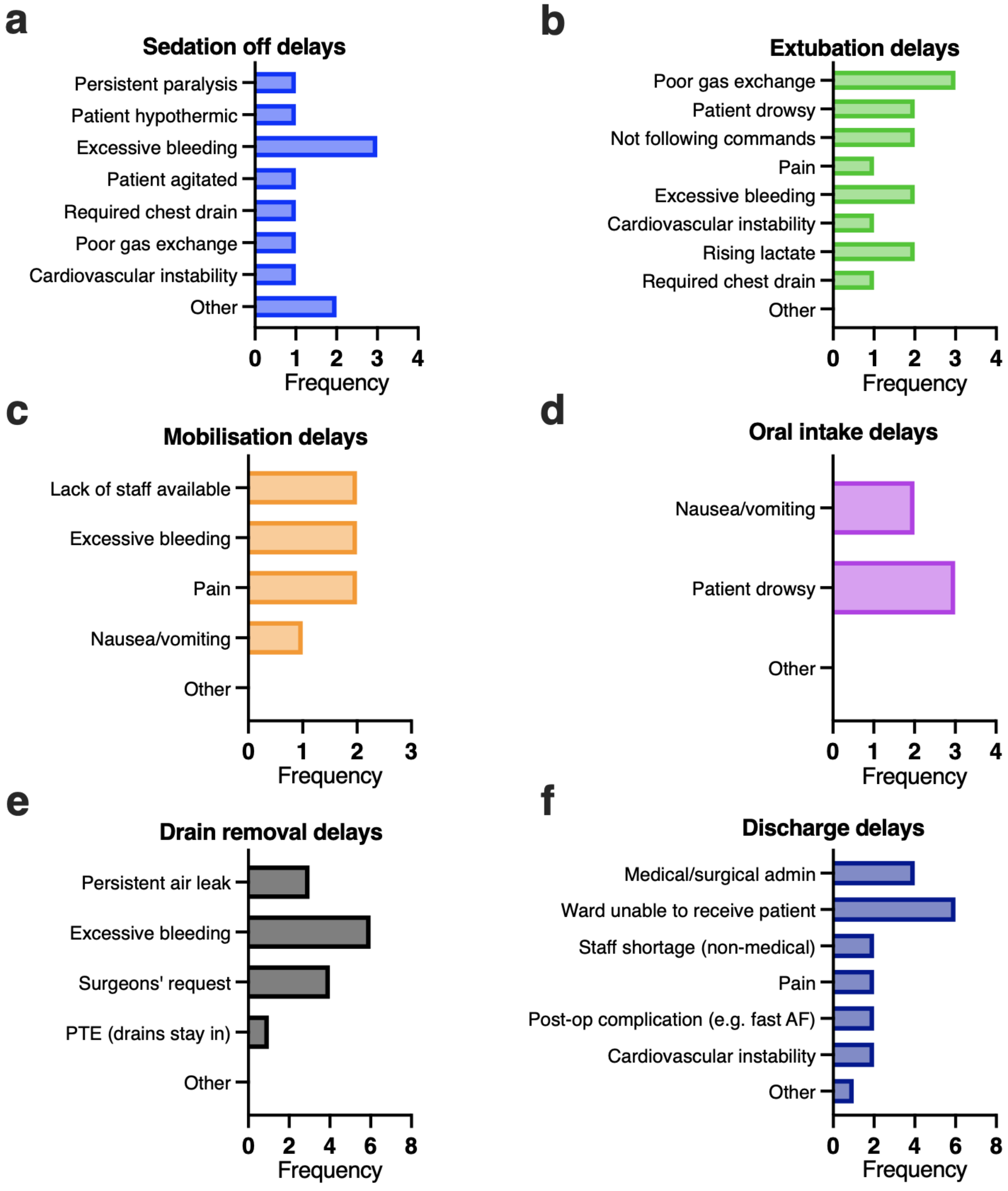

Supplementary Figure 1| Categorisation of reasons reported for delays in meeting each key performance indicator in PDSA 1. Reasons for delay were examined and categorised as shown in panels **a–f**. Note that in some cases, multiple reasons may have contributed to an overall delay in meeting each KPI. Abbreviations: PTE = pulmonary thromboendarterectomy, AF = atrial fibrillation
