# Supplementary material for: Improving implementation of Enhanced Recovery After Surgery (ERAS) to increase timeliness of recovery after cardiac surgery: a quality improvement project
Source: BMJ Open Qual. 2026 Feb 2;15(1):e003612. doi: 10.1136/bmjoq-2025-003612 (PMC12878191; doi:10.1136/bmjoq-2025-003612)
Supplement: online supplemental figure 2 [file bmjoq-15-1-s002.pdf]

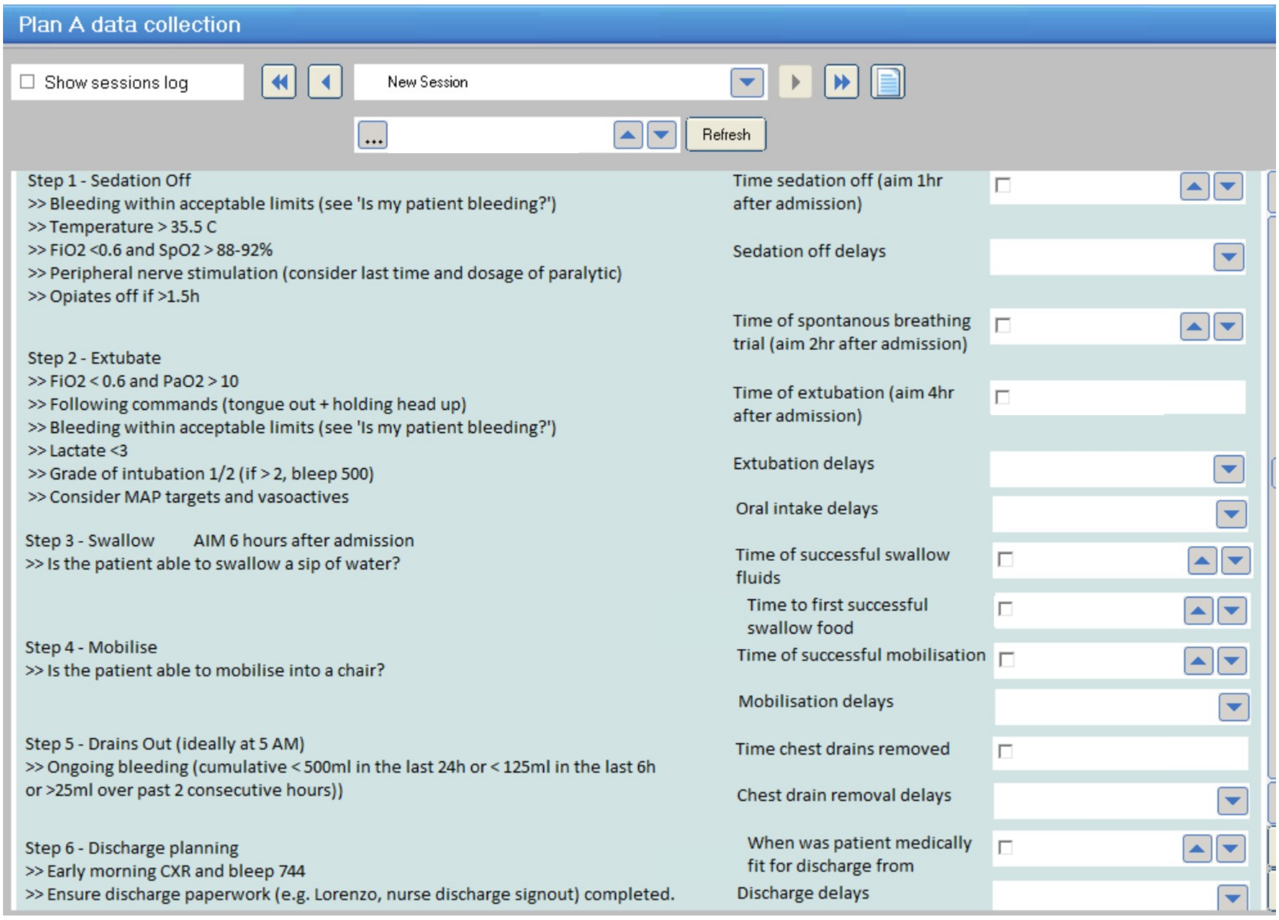

Supplementary Figure 2| Data collection tool deployed in the electronic patient record. This was introduced as a mandatory element in the EPR as part of PDSA-2 for elective surgical patients, allowing extraction of data at monthly intervals for ongoing analysis.
