# Supplementary material for: Improving implementation of Enhanced Recovery After Surgery (ERAS) to increase timeliness of recovery after cardiac surgery: a quality improvement project
Source: BMJ Open Qual. 2026 Feb 2;15(1):e003612. doi: 10.1136/bmjoq-2025-003612 (PMC12878191; doi:10.1136/bmjoq-2025-003612)
Supplement: online supplemental figure 3 [file bmjoq-15-1-s003.pdf]

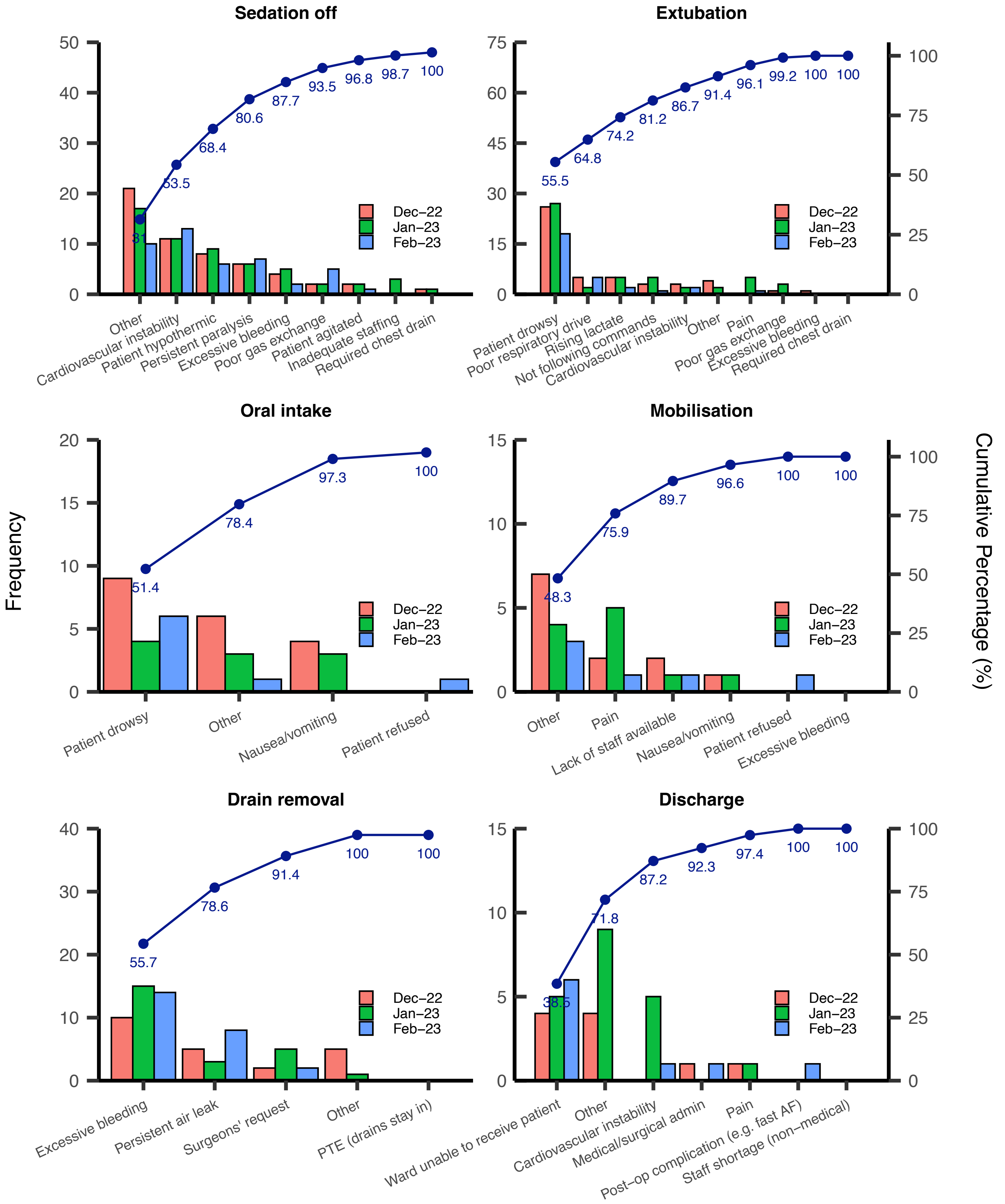

Supplementary Figure 3| Pareto charts demonstrating reasons for delays across PDSA-2. Reasons for delays across each KPI are listed in descending order of frequency. Bars represent month-by-month breakdown of frequency of reported delays via the EPR tool. The Pareto line (dark blue) tracks the cumulative percentage of the reasons for delays.
