# Supplementary material for: Improving implementation of Enhanced Recovery After Surgery (ERAS) to increase timeliness of recovery after cardiac surgery: a quality improvement project
Source: BMJ Open Qual. 2026 Feb 2;15(1):e003612. doi: 10.1136/bmjoq-2025-003612 (PMC12878191; doi:10.1136/bmjoq-2025-003612)
Supplement: online supplemental table 1 [file bmjoq-15-1-s004.pdf]

<table>
<tr><th>KPI</th><th>Objective delays</th><th>Reported delays</th></tr>
<tr><td>Sedation off</td><td>32/34</td><td>10/34</td></tr>
<tr><td>SBT</td><td>29/34</td><td>30/34</td></tr>
<tr><td>Extubation</td><td>30/34</td><td>13/34</td></tr>
<tr><td>Oral intake</td><td>27/33</td><td>4/33</td></tr>
<tr><td>Mobilisation</td><td>29/30</td><td>1/30*</td></tr>
<tr><td>Drains out</td><td>21/29</td><td>18/29*</td></tr>
<tr><td>Ward-ready</td><td>5/29</td><td rowspan="2">18/28**</td></tr>
<tr><td>Discharged</td><td>13/33</td></tr>
</table>

** of which 1 was not an actual delay*

*** of which 3 were not actual delays* and *had a discharge latency <1 h*

Supplementary Table 1| Objective vs. nurse-reported delays for each KPI in PDSA 1. Note that not all forms were completely filled in by the bedside nurses, hence n≠34 in several instances in this table.
